# Diagnostic evaluation of urea nitrogen/creatinine ratio in dogs with gastrointestinal bleeding

**DOI:** 10.1111/jvim.16101

**Published:** 2021-03-17

**Authors:** Jenny Stiller, Alice M. Defarges, Brigitte A. Brisson, Alexa M. E. Bersenas, Jill S. Pomrantz, Brittany Lang, David L. Pearl

**Affiliations:** ^1^ Department of Clinical Studies, Ontario Veterinary College University of Guelph Guelph Ontario Canada; ^2^ Small Animal Clinic, College of Veterinary Medicine University of Leipzig Leipzig Saxony Germany; ^3^ North America Medical Consulting Services IDEXX Laboratories, Inc. Westbrook Maine USA; ^4^ Department of Population Medicine, Ontario Veterinary College University of Guelph Guelph Ontario Canada

**Keywords:** anemia, BUN creatinine ratio, canine, gastroenterology, hemorrhage, video capsule endoscopy

## Abstract

**Background:**

Urea nitrogen/creatinine ratio (UCR) is a marker for upper gastrointestinal bleeding (GIB) in people.

**Objectives:**

To assess the usefulness of UCR to predict occult GIB and distinguish upper from lower GIB in dogs.

**Animals:**

Eighty‐nine dogs with GIB and 65 clinically healthy dogs. Dogs were grouped according to 65 overt GIB and 24 occult GIB, and based on lesion localization (37 upper, 13 lower, and 8 both).

**Methods:**

Seventy‐four dogs were included retrospectively and 15 dogs prospectively. Serum urea nitrogen and creatinine concentrations, UCR, hemoglobin concentration, hematocrit, mean corpuscular volume, and mean corpuscular hemoglobin concentration were compared between groups. Logistic regression models were fitted to assess if variables could distinguish occult GIB from being healthy and upper from lower GIB.

**Results:**

The UCR was significantly higher in dogs with overt GIB compared to control dogs (*P* = .02) and dogs with occult GIB (*P* = .05). The UCR was not significantly associated with occult GIB vs being healthy, or upper vs lower GIB (*P* > .05 each). Dogs with higher hemoglobin concentration and hematocrit had significantly lower odds of having occult GIB than being healthy (*P* < .0001 each).

**Conclusions and Clinical Importance:**

The UCR does not seem to be a clinically useful marker of occult GIB and appears to have poor discriminatory ability between upper and lower GIB. An increased UCR in a dog without signs of overt GIB, especially if its hematocrit is within the middle or upper reference interval, does not appear to warrant prompt prescription of gastrointestinal protectants.

**Abbreviations**
ANOVA: analysis of variance
CI: confidence interval
GI: gastrointestinal
GIB: gastrointestinal bleeding
Hb: hemoglobin
Hct: hematocrit
MCHC: mean corpuscular hemoglobin concentration
MCV: mean corpuscular volume
n: sample size
OVC HSC: Ontario Veterinary College Health Sciences Centre
UCR: urea nitrogen/creatinine ratio
VCE: video capsule endoscopy

## 1 INTRODUCTION

Gastrointestinal bleeding (GIB) is a frequent cause of hospitalization in dogs.^1^, ^2^, ^3^ Clinical signs vary from subclinical disease without visible bleeding (occult GIB) to visible hemorrhage (overt GIB) including hematemesis, hematochezia, and melena.^3^, ^4^ Gastrointestinal bleeding can further be distinguished into upper and lower gastrointestinal (GI) hemorrhage based on the location of bleeding either orad or aborad to the ligament of Treitz (duodenojejunal junction).^5^ To properly manage and treat GIB, accurate identification of the source of GI hemorrhage is required.

The ratio of serum urea nitrogen to creatinine concentration has been used as a simple index to discriminate upper (higher ratio) from lower GIB (lower ratio) sources in human medicine.^5^, ^6^, ^7^ Different urea nitrogen/creatinine ratio (UCR) cutoffs to differentiate upper from lower GIB ranging from 30 to 36 have been reported in people.^7^, ^8^, ^9^, ^10^, ^11^ Dogs with melena, hematemesis, or both had significantly higher UCR than did control dogs.^1^ It is postulated that an increase in serum urea concentration in upper GIB is caused by increased hepatic ureagenesis after metabolism of blood proteins in the GI tract.^6^, ^12^, ^13^ Experimental studies in people and dogs showed an association between ingestion of blood and increased serum urea concentration.^12^, ^13^, ^14^ An alternative hypothesis is that early prerenal azotemia associated with blood loss and subsequent hypovolemia causes increased serum urea concentration without an increase in serum creatinine concentration.^14^, ^15^ To our knowledge, the diagnostic value of the index to predict occult GIB and localize GIB has not been investigated in dogs.

Our primary objective was to evaluate the usefulness of the UCR as a diagnostic marker for occult GIB and to distinguish upper from lower GIB in dogs. We hypothesized that UCR would be higher in dogs with occult GIB compared to healthy dogs but lower compared to dogs with overt GIB, and that UCR would predict occult GIB. Moreover, we anticipated that UCR would be higher in dogs with upper vs lower GIB, and that the ratio could predict upper GIB. As a secondary objective, we compared the diagnostic value of UCR to routine hematological variables in dogs with GIB.

## 2 MATERIALS AND METHODS

### 2.1 Patient population

This project was a multicenter, observational, retrospective, and prospective study. Seventy‐four dogs were included retrospectively and 15 dogs prospectively. For retrospective enrollment, electronic medical records of the Ontario Veterinary College Health Sciences Centre (OVC HSC, University of Guelph, Canada) between July 2011 and July 2017 were reviewed. Additional records of dogs that had received video capsule endoscopy (VCE; ALICAM, Infiniti Medical, Redwood City, CA) between July 2015 and July 2017 were provided by Infiniti Medical. These patients were presented to veterinarians for GI‐related clinical signs or anemia of unknown origin.

Dogs were enrolled prospectively from August 2017 to March 2020 as part of a separate prospective study concerning VCE. The study protocol was approved by the University of Guelph Animal Care Committee. Owner consent was obtained before study enrollment. Dogs with VCE‐documented GIB presented to 1 of the following veterinary referral hospitals: OVC HSC, Mississauga Oakville Veterinary Emergency and Specialty Hospital (Oakville, Canada), and Veterinary Emergency Clinic (Toronto, Canada) were eligible for inclusion based on clinical signs of overt GIB or suspected occult GIB. These dogs received VCE by ALICAM PO or by endoscopic placement into the stomach or duodenum. Dogs with body weight of <4.5 kg, coagulopathy, suspected partial or complete GI obstruction, or GI perforation were excluded from VCE examination.

For all identified eligible dogs (retrospective and prospective case enrollment), the medical records were reviewed and the following exclusion criteria applied: presentation in shock, moderate or marked dehydration at admission based on physical examination, signs of hemoconcentration (hematocrit [Hct] > 0.6 L/L) or both, suspected or confirmed renal azotemia (serum creatinine concentration >2 mg/dL and, if available, urine specific gravity <1.030), postrenal azotemia, signs of hepatic dysfunction (as determined by serum biochemical test results, and if available fasted blood ammonia concentration, pre‐ and postprandial serum bile acids concentration, coagulation panel, abdominal ultrasonography or some combination of these), documented marked muscle mass loss, and pyrexia. Patients were also excluded if occult GIB could not be confirmed or if temporally relevant serum urea nitrogen and creatinine concentrations were not available (ie, at admission when overt GIB was present or within 72 hours of diagnosis of occult GIB). Dogs with corticosteroid exposure initially were included for further analysis given previous contradictory study results on the influence of corticosteroid exposure on UCR in dogs with GIB.^1^, ^16^

### 2.2 Control dogs

Sixty‐five healthy control dogs with available blood test results (CBC, biochemical profile) were selected. These dogs were presented to the OVC HSC for blood donation, for elective castration or spay, or were healthy dogs participating in an unrelated research study. Control dogs were deemed healthy based on history, physical examination findings, and clinically unremarkable CBC and serum biochemistry profile results. Control dogs were excluded if anorexia, vomiting, diarrhea, pica, regurgitation, or abdominal discomfort were present within the last 2 weeks or if ulcerogenic drugs had been administered within the last 4 weeks. Two dogs had received phenylpropanolamine and 1 dog had received diethylstilbestrol for urethral sphincter mechanism incompetence, and 1 dog had received trazodone before blood collection. No other medications besides preventative flea and tick medication had been administered.

### 2.3 Data collection

For all eligible dogs, medical records were reviewed for the following data: signalment, history, clinical signs, medications at the time of presentation, results of blood tests, results of diagnostic tests to identify bleeding lesions, final diagnosis, and survival to discharge. For each dog enrolled prospectively, muscle condition and systemic blood pressure at the time of blood collection were recorded. Only temporally relevant blood test results were included.

All laboratory tests were performed by reference laboratories (Animal Health Laboratory, University of Guelph, Guelph, Canada; IDEXX Reference Laboratories Ltd., Antech Diagnostics Reference Laboratories) or using standard in‐house analyzers. Information on biochemistry and hematology analyzers used at the reference laboratories is given in Table S1 and Table S2. The biochemistry analyzers all measured the entire molecule urea using the kinetic test method.^17^, ^18^, ^19^ Because urea concentration and its ratio to creatinine concentration traditionally are reported using serum urea nitrogen concentration in mg/dL, we converted urea concentrations reported in international units (mmol/L) to urea nitrogen concentrations in conventional units (mg/dL) by dividing the result by 0.357.^20^ If serum creatinine concentrations were reported in international units (μmol/L), conversion to conventional units (mg/dL) was performed by dividing the result by 88.4.^20^

All dogs included in further analyses were subclassified. Gastrointestinal bleeding was defined as all forms of hemorrhage in the digestive tract extending from the oral cavity to the rectum. Dogs were defined as overt GI bleeders if melena, hematemesis, or hematochezia were present. Dogs classified as occult GI bleeders did not have visible signs of GIB, and GI hemorrhage was confirmed on diagnostic evaluation. Dogs were classified as having upper GIB, lower GIB, or both based on the bleeding source being orad, aborad, or both relative to the ligament of Treitz, respectively.

### 2.4 Tests to diagnose GIB

The diagnostic evaluation performed to identify bleeding GI lesions varied and included at least 1 of the following: conventional GI endoscopy, VCE, exploratory laparotomy, or necropsy. For all dogs, conventional endoscopy (upper or lower GI endoscopy or both) was performed and assessed by a board‐certified internist or a resident in internal medicine. All VCE examinations were performed after a 12‐ to 24‐hour fast, and capsules were administered PO or endoscopically. The capsules were evaluated by a single board‐certified internist (retrospective part: JP, Infiniti Medical; prospective part: AD). Gastrointestinal bleeding was diagnosed on endoscopy (traditional or VCE) if an actively or recently bleeding lesion was identified. Exploratory laparotomy was performed by a board‐certified surgeon at the OVC HSC. Bleeding lesions were identified if GI ulcers or bleeding masses were visualized. Necropsies were performed by a board‐certified pathologist at the Animal Health Laboratory (University of Guelph, Guelph, Canada).

### 2.5 Statistical analyses

Sample size calculation was performed based on a pilot study including retrospective data collected from 15 dogs with occult GIB, for which we had not applied equally stringent exclusion criteria, and 17 dogs with no GIB. The median value of UCR (ie, 20) among these dogs was used as a cutoff to differentiate high vs low UCR. Based on this cutoff, 66.7% of dogs with occult GIB had high UCR and 35.3% of dogs with no GIB had high UCR. Based on recruitment of 30 dogs with occult GIB and 30 dogs without GIB, we would have had 80% power to detect a significant difference with a level of significance of .05 between the 2 groups.

Descriptive statistics were reported for all variables. Categorical variables were presented as frequencies or percentages. Numerical data were tested for normality using the Shapiro‐Wilk test and inspection of QQ plots. Normally distributed data were expressed as mean ± SD. Non‐normally distributed data were expressed as median and range. Chi‐squared or Fisher's exact tests were used for comparisons of proportions of sex and neuter status between overt and occult GI bleeders and clinically healthy dogs.

To compare serum urea nitrogen and creatinine concentrations, and UCR between GI bleeders with and without corticosteroid administration, independent *t*‐tests with logarithmically transformed data to meet test assumptions were performed. Dogs that had received corticosteroids were excluded from the remaining statistical analyses if a statistically significant difference was identified for serum urea nitrogen or creatinine concentration or UCR between them and dogs not treated with corticosteroids.

To assess if the variables serum urea nitrogen concentration, serum creatinine concentration, UCR, hemoglobin (Hb), Hct, mean corpuscular volume (MCV), and mean corpuscular hemoglobin concentration (MCHC) were statistically different between groups (overt GIB vs occult GIB vs healthy; upper GIB vs lower GIB vs both), 1‐way analysis of variance (ANOVA) or, if its assumptions were not met, Kruskal‐Wallis tests were performed. In the event of significant test results, Tukey‐Kramer (ANOVA) or Dunn‐Bonferroni (Kruskal‐Wallis) comparison post hoc tests were performed to identify significant differences between groups. Non‐normally distributed data were logarithmically transformed if necessary to meet underlying statistical assumptions.

To identify predictors of occult GIB and upper GIB, logistic regression models were constructed. Serum urea nitrogen and creatinine concentrations, UCR, Hb, Hct, MCV, and MCHC each were included as explanatory variables. Presence of occult GIB (occult vs healthy) and presence of upper GIB (upper GIB vs lower GIB) were included as dependent variables. The assumption of linearity was examined by including a quadratic term for each laboratory variable. If the *P* value for the quadratic term was significant, the variable was categorized into quartiles because the assumption of linearity was not met.

The potential confounding effect of several variables was assessed by including these as covariates in the models. The following variables were included for assessment of a confounding or distorting effect: age (assessed for all independent variables), weight (assessed for serum creatinine concentration and UCR), presence of weight loss or anorexia (assessed for serum urea nitrogen and creatinine concentrations and UCR), and presence of overt vs occult GIB (assessed for all independent variables in the model to predict upper GIB). If the inclusion of these covariates resulted in a ≥20% change in the coefficient of the independent variable being explored, we reported the adjusted odds ratio; otherwise only univariable results were reported.

The fit of models was assessed using the Hosmer‐Lemeshow or Pearson goodness‐of‐fit tests depending on whether the data were binary or binomial.^21^ Scatter plots of residuals and predicted values of logistic regression models were used to identify outliers.

Commercial statistical software packages (MedCalc Statistical Software 18.11.6, MedCalc Software bvba, Ostend, Belgium; R Statistical Software 3.6.2, R Foundation for Statistical Computing, Vienna, Austria) were used for all analyses. Significance level was set .05.

## 3 RESULTS

One‐hundred seventeen dogs with GIB were identified for inclusion in the study, 28 were receiving corticosteroids, and 89 were not. Dogs receiving glucocorticoids had significantly lower serum creatinine concentration (median, 0.62 mg/dL; range, 0.29‐1.57 mg/dL) and significantly higher UCR (median, 27.8; range, 8‐73.6) compared to dogs that had not received corticosteroids (creatinine, 0.85 mg/dL; range, 0.20‐1.92 mg/dL; *P* = .04; UCR, 18.2; range, 7.2‐89.4; *P* = .01). No statistically significant difference in serum urea nitrogen concentration was noted between dogs receiving corticosteroids (median, 17.7 mg/dL; range, 7.3‐42.3 mg/dL) and those that did not (median, 15.7 mg/dL; range, 4.2‐71.0 mg/dL, *P* = .31). Given the significant difference in serum creatinine concentration and UCR, dogs receiving corticosteroids were excluded from the study. No dogs were diagnosed with hyperadrenocorticism.

### 3.1 Study population

**FIGURE 1:**
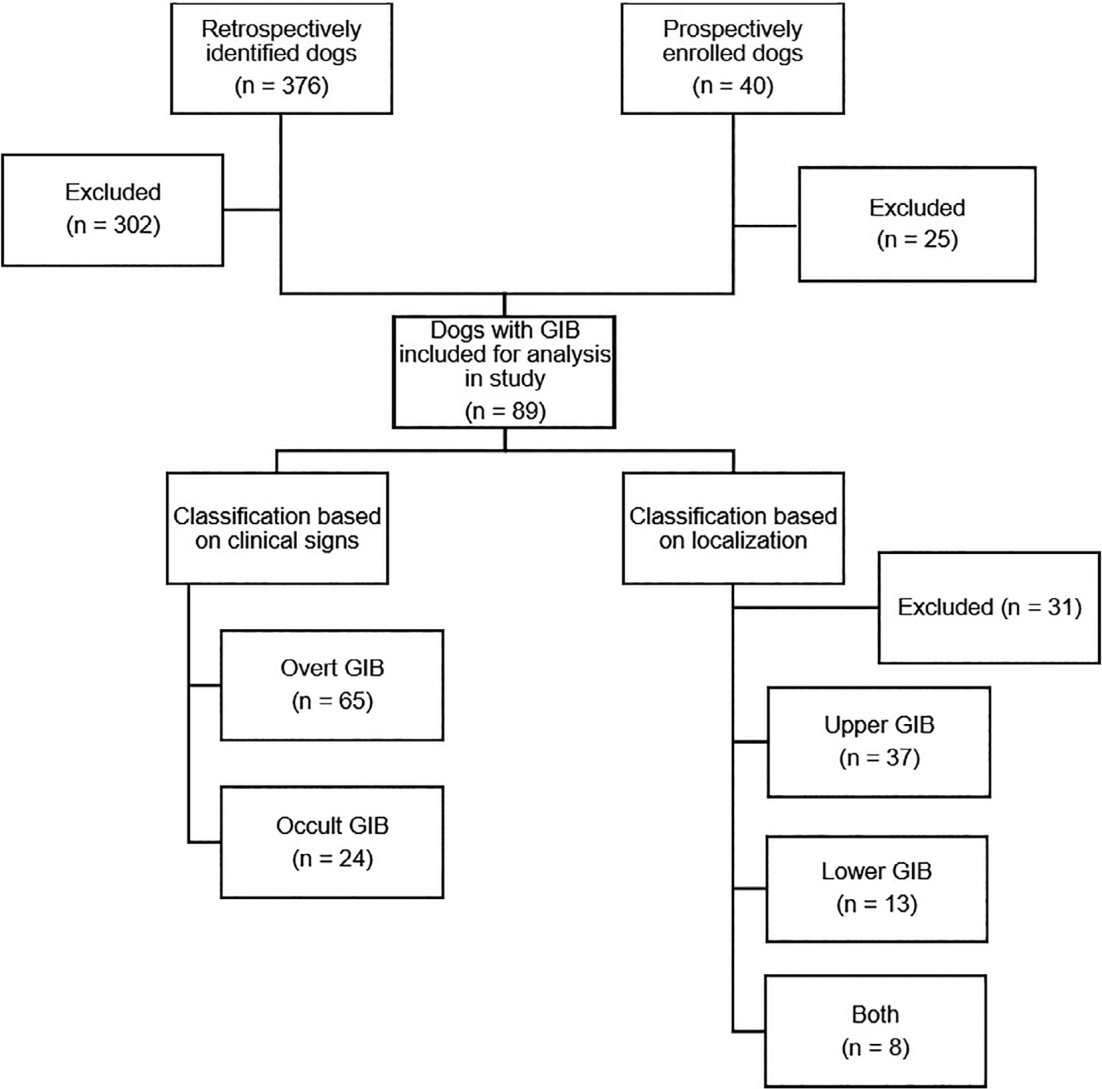
Flow chart summarizing the number of dogs with gastrointestinal bleeding (GIB) included in the analysis and the classification based on clinical signs and on localization of the bleeding lesion. Three hundred seventy‐six dogs were identified retrospectively. Of these, 302 cases were excluded from the analysis for the following reasons: hemorrhage not confirmed in cases of possible occult GIB (156), missing serum urea nitrogen and creatinine concentrations at time of clinical signs (overt GIB) or within 72 hours of diagnosis of occult GIB (74), presentation in shock and/or moderate or marked dehydration (31), administration of corticosteroids (28), hepatic dysfunction (5), renal/postrenal azotemia (5), and pyrexia (3). Forty dogs were prospectively enrolled. Of these, 25 dogs were excluded for the following reasons: no bleeding lesions identified (15), administration of corticosteroids (8), hepatic dysfunction (1), and marked muscle mass loss (1)

In total, 89 dogs with GIB were included in our analyses. There were 65 dogs with overt and 24 dogs with occult GIB (Figure 1). Sixty‐five clinically healthy dogs were included. Of these, 37 dogs were blood donors, 19 dogs were part of an unrelated research study, and 9 dogs were presented for elective castration or spay. Signalment and clinical signs of overt GI bleeders, occult GI bleeders, and control dogs are presented in Tables 1 and 2, respectively. Control dogs were significantly younger compared to overt GI bleeders (Table 1). Of all dogs with GIB and control dogs, 56 breeds were represented, with dogs of mixed breed (39; 43.8%), Labrador Retriever (13; 14.6%), and Golden Retriever (12; 13.5%) being most frequently represented. Of the 15 dogs enrolled prospectively, 2 were documented to have mild muscle loss and all dogs were normotensive at the time of blood collection.

**TABLE 1:** Signalment of dogs with overt and occult GIB and clinically healthy dogs

	Overt GIB	Occult GIB	Healthy	*P* value
Total number, n	65	24	65	—
Age in years, median (range)	8 (0.2‐14)^a^	7 (0.8‐12)	4 (0.5‐11)^a^	<.0001^b^
Body weight in kg, median (range)	18.9 (2.9‐60)	22.0 (4.2‐67)	28.5 (3‐66.5)	.12^b^
Sex (female/male)	29/36	11/13	32/33	.87^c^
Neuter status (neutered/intact)	59/6	19/5	52/13	.18^c^

Abbreviation: GIB, gastrointestinal bleeding.

^a^ Post hoc test revealed significant difference between the groups marked with the same letter.

^b^ Kruskal‐Wallis test.

^c^ Chi‐squared test.

**TABLE 2:** Clinical signs of dogs with overt and occult GIB

	Overt GIB, n (%)	Occult GIB, n (%)
Total number, n	65	24
GI‐related clinical signs		
Anorexia	36 (55.4%)	13 (54.2%)
Vomiting	36 (55.4%)	9 (37.5%)
Diarrhea	30 (46.2%)	8 (33.3%)
Lethargy	33 (50.8%)	8 (33.3%)
Weight loss	12 (18.5%)	9 (37.5%)
Abdominal pain	50 (76.9%)	15 (23.1%)
Pica	6 (9.2%)	3 (12.5%)
Regurgitation	3 (4.6%)	2 (8.3%)
Clinical signs of GIB		
Hematemesis	23 (35.4%)	—
Melena	35 (53.8%)	—
Hematochezia	29 (44.6%)	—

Abbreviation: GIB, gastrointestinal bleeding.

In 58 dogs with GIB (34 overt GIB, 24 occult GIB), additional diagnostic tests were performed and allowed localization of bleeding along the GI tract (Figure 1). Thirty‐seven (63.8%) dogs had upper GIB, 13 (22.4%) had lower GIB, and 8 (13.8%) had both. Signalment and clinical signs of dogs with bleeding of the upper and lower GI tract are presented in Tables 3 and 4, respectively. Most dogs with lower GIB had overt clinical signs, whereas almost half of the dogs with upper GIB had occult bleeding. The underlying causes of GIB identified in these 89 dogs were erosive or ulcerative lesions of different etiology (61; 68.5%), coagulopathies (7; 7.9%), and vascular ectasia (2; 2.2%). In 19 (21.3%) dogs, the cause of GIB was not identified. The most common coagulopathy was immune‐mediated thrombocytopenia; 1 dog was diagnosed with von Willebrand disease. Thirty‐eight (42.7%) dogs had received GI protectants at the time of admission to the hospital or before VCE. Two (2.3%) dogs with GIB were euthanized because of the severity of clinical signs and suspicion of underlying neoplasia.

**TABLE 3:** Signalment of dogs with GIB in the upper, lower, and both parts of the digestive system

	Upper GIB	Lower GIB	Both	*P* value
Total number, n	37	13	8	—
Age in years, median (range)	8 (0.6‐12)	8 (0.8‐12)	9 (2‐11)	.50^a^
Body weight in kg, median (range)	22.7 (4‐67)	21.4 (2.9‐38)	18.6 (11‐46)	.74^a^
Sex (female/male)	19/18	5/8	4/4	.74^b^
Neuter status (neutered/intact)	30/7	12/1	7/1	.86^b^

*Note:* The diagnostic tests used to identify the causes of bleeding were the following: VCE (27 dogs; 46.6%), esophagogastroscopy or esophagogastroduodenoscopy (17; 29.3%), bidirectional GI endoscopy (4; 6.9%), exploratory laparotomy (3; 5.2%), ileocolonoscopy or colonoscopy (3; 5.2%), rectal or oral examination (3; 5.2%), and necropsy (1; 1.7%). Six dogs that underwent VCE had an incomplete study defined as failure to reach the colon during recording time, but images from the stomach showed bleeding gastric lesions.

Abbreviations: GIB, gastrointestinal bleeding; VCE, video capsule endoscopy.

^a^ Kruskal‐Wallis test.

^b^ Fisher's exact test.

**TABLE 4:** Clinical signs in dogs with GIB in the upper, lower, and both parts of the digestive system

	Upper GIB, n (%)	Lower GIB, n (%)	Both, n (%)
Total number, n	37	13	8
GI‐related clinical signs			
Anorexia	22 (59.5%)	4 (30.8%)	5 (62.5%)
Vomiting	21 (56.8%)	1 (7.7%)	4 (50%)
Diarrhea	13 (35.1%)	7 (53.8%)	5 (62.5%)
Lethargy	15 (40.5%)	3 (23.1%)	2 (25%)
Weight loss	15 (40.5%)	1 (7.7%)	4 (50%)
Abdominal pain	10 (27.0%)	3 (23.1%)	2 (25%)
Clinical signs of GIB			
Overt GIB	17 (45.9%)	10 (76.9%)	7 (87.5)
Hematemesis	2 (5.4%)	0	0
Melena	5 (13.5%)	0	2 (25%)
Hematochezia	4 (10.8%)	7 (53.8%)	1 (12.5%)
Hematemesis and melena	3 (8.1%)	0	0
Hematemesis and hematochezia	1 (2.7%)	0	2 (25%)
Melena and hematochezia	2 (5.4%)	3 (23.1%)	2 (25%)
Occult GIB	20 (54.1%)	3 (23.1%)	1 (12.5%)

Abbreviation: GIB, gastrointestinal bleeding.

### 3.2 UCR and hematological variables in dogs with overt and occult GIB and clinically healthy dogs

Descriptive statistics and comparison testing for serum urea nitrogen and creatinine concentrations and UCR in dogs with overt and occult GIB and clinically healthy dogs are presented in Table 5. Serum urea nitrogen concentration was significantly lower in dogs with occult GIB compared to control dogs and dogs with overt GIB. Serum creatinine concentration in dogs with overt and occult GIB was significantly lower compared to healthy control dogs. In dogs with overt GIB compared to control dogs and dogs with occult GIB, UCR was significantly higher.

**TABLE 5:** Comparison of serum urea nitrogen and creatinine concentrations, UCR, Hb, Hct, MCV, and MCHC between dogs with overt and occult GIB and clinically healthy dogs

	Overt GIB	Occult GIB	Healthy	*P* value
Urea nitrogen (mg/dL)	17.6 (4.2‐71)^c^	12.3 (5.6‐44.5)^c,d^	17.4 (9.2‐29.4)^d^	.03^a^
Creatinine (mg/dL)	0.89 ± 0.31^c^	0.82 ± 0.28^d^	1.02 ± 0.24^c,d^	.002^b^
UCR	19.4 (8.1‐89.4)^c,d^	15.1 (7.2‐75.7)^c^	16.2 (8.0‐47.7)^d^	.008^a^
Hb (g/L)	136.2 ± 46.2^c^	122.05 ± 42.2^d^	174.5 ± 17.09^c,d^	<.0001^a^
Hct (L/L)	0.43 (0.13‐0.60)^c^	0.38 (0.11‐0.53)^d^	0.51 (0.42‐0.65)^c,d^	<.0001^a^
MCV (fL)	70 (54‐83)	71 (38‐89)	71 (64‐78)	.31^a^
MCHC (g/L)	334 (253‐372)^c^	337 (277‐396)	340 (323‐366)^c^	.01^a^

*Note:* Sixty‐five dogs had overt and 24 dogs had occult GIB; 65 dogs were clinically healthy. The non‐normally distributed data are expressed as median (range). Normally‐distributed data were expressed as mean ± standard deviation. For dogs with overt GIB, Hb, MCV, and MCHC results were available for 62 dogs, and Hct results were available for 63 dogs. For dogs with occult GIB, Hb and MCHC were available for 21, Hct and MCV for 22 dogs.

Abbreviations: GIB, gastrointestinal bleeding; Hb, hemoglobin; Hct, hematocrit; MCHC, mean corpuscular hemoglobin concentration; MCV, mean corpuscular volume; UCR, urea nitrogen/creatinine ratio.

^a^ Kruskal‐Wallis test.

^b^ ANOVA.

^c/d^ Post hoc test revealed significant difference between the groups marked with the same letter.

Serum urea nitrogen and creatinine concentrations were significantly associated with the odds of having occult GIB compared to being clinically healthy, but the UCR was not significantly associated with this outcome (Table 6). Dogs with serum urea nitrogen concentration of ≤12 mg/dL (first quartile) were significantly more likely to have occult GIB than were dogs with serum urea nitrogen concentration ranging from >12 to ≤21.5 mg/dL (second and third quartiles, Table 6). The odds of occult GIB were lower in dogs with higher serum creatinine concentrations (Table 6). Urea nitrogen/creatinine ratio was not a statistically significant variable, even after controlling for the confounding effects of weight loss and anorexia. For each of the logistic regression models used, no evidence of lack of model fit was found and no outliers were identified.

**TABLE 6:** Results of logistic regression models examining the association of serum urea nitrogen and creatinine concentrations, UCR, Hb, Hct, MCV, and MCHC and the odds of having occult GIB in dogs compared to being clinically healthy

	n	Odds ratio	95% CI	*P* value
Urea nitrogen (mg/dL)	89	—	—	
1. Quartile (5 > Urea nitrogen ≤12)	22	(Referent)	—	—
2. Quartile (12 > Urea nitrogen ≤16)	25	0.22^a^	0.05‐0.99	.05
3. Quartile (16 > urea nitrogen ≤21.5)	21	0.07^a^	0.01‐0.66	.02
4. Quartile (21.5 > urea nitrogen ≤45)	21	0.26^a^	0.06‐1.19	.08
Creatinine (mg/dL)	89	0.04	0.01‐0.33	.003
UCR	89	1.02^a^	0.97‐1.07	.45
		1.02^b^	0.95‐1.09	.60
Hb (g/L)	86	0.91	0.87‐0.95	.0001
Hct (L/L) per 100 units	87	0.70	0.58‐0.84	.0001
MCV (fL)	87	—	—	
1. Quartile (37 > MCV ≤68)	22	(Referent)	—	—
2. Quartile (68 > MCV ≤71)	28	0.58^c^	0.16‐2.11	.41
3. Quartile (71 > MCV ≤73)	20	0.21^c^	0.04‐1.06	.06
4. Quartile (73 > MCV ≤89)	17	1.14^c^	0.29‐4.49	.85
MCHC (g/L)	86	—	—	
1. Quartile (276 > MCHC ≤332)	22	(Referent)	—	—
2. Quartile (332 > MCHC ≤339)	21	0.73^c^	0.19‐2.53	.59
3. Quartile (339 > MCHC ≤346)	26	0.26^c^	0.05‐1.01	.06
4. Quartile (346 > MCHC ≤396)	17	0.64^c^	0.15‐2.78	.55

*Note:* Twenty‐four dogs had occult GIB; 65 dogs were clinically healthy. For dogs with occult GIB, Hb and MCHC were available for 21 and Hct and MCV for 22 dogs. Serum urea nitrogen concentration, MCV, and MCHC were modeled as categorical variable to meet the assumption of linearity. Confounding effect of age was assessed for all variables, the effect of weight was assessed for serum creatinine concentration and UCR, and the presence of anorexia and weight loss for serum urea nitrogen and creatinine concentrations, and UCR. If the inclusion of the covariates resulted in a ≥20% change in the coefficient of the independent variable, the adjusted odds ratio was reported.

Abbreviations: CI, confidence interval; GIB, gastrointestinal bleeding; Hb, hemoglobin; Hct, hematocrit; MCHC, mean corpuscular hemoglobin concentration; MCV, mean corpuscular volume; n, sample size; UCR, urea nitrogen/creatinine ratio.

^a^ Adjusted odds ratio after inclusion of presence of weight loss as covariate.

^b^ Adjusted odds ratio after inclusion of presence of anorexia as covariate.

^c^ Adjusted odds ratio after inclusion of age as covariate.

Hemoglobin concentration and Hct were significantly lower in dogs with overt and occult GIB compared to healthy dogs (Table 5). Mean corpuscular volume was not significantly different between groups (Table 5). Overt GI bleeders had significantly lower MCHC compared to healthy dogs (Table 5).

Using logistic regression models, dogs with higher Hb concentration and Hct had significantly lower odds of occult GIB (Table 6). No significant association was found between MCV or MCHC and the odds of having occult GIB after adjusting for age (Table 6).

### 3.3 UCR and hematological variables in dogs with upper and lower GIB

Descriptive statistics and results of comparison testing for serum urea nitrogen and creatinine concentrations, and UCR in dogs with upper GIB, lower GIB, and hemorrhage at both sites are presented in Table 7. No significant difference was found between these groups for serum urea nitrogen and creatinine concentrations and UCR.

**TABLE 7:** Comparison of serum urea nitrogen and creatinine concentrations, UCR, Hb, Hct, MCV, and MCHC in dogs with upper GIB, lower GIB and with both upper and lower GIB

	Upper GIB	Lower GIB	Both	*P* value^a^
Urea nitrogen (mg/dL)	15.7 (5.6‐71.0)	18.8 (9.5‐51.5)	17.9 (10.6‐35.8)	.31
Creatinine (mg/dL)	0.81 ± 0.31	0.86 ± 0.39	0.93 ± 0.19	.42
UCR	17.0 (7.2–75.7)	22.41 (10.4‐36.3)	19.2 (11.7‐37.3)	.67
Hb (g/L)	141.5 (35‐201)	155 (43‐192)	118.5 (36‐202)	.95
Hct (L/L)	0.4 ± 0.11	0.38 ± 0.15	0.39 ± 0.17	.99
MCV (fL)	70 (38‐81)	69 (41.7‐88.6)	73 (58‐79)	.73
MCHC (g/L)	333 ± 21	334 ± 28	317 ± 30	.34

*Note:* Thirty‐seven dogs were diagnosed with upper and 13 with lower GIB; 8 dogs had both upper and lower GIB. Upper GIB was defined as hemorrhage orad to the ligament of Treitz (duodenojeunal junction). The non‐normally distributed data are expressed as median (range). Normally distributed data were expressed as mean ± SD. Results for Hb and MCHC were available from 53 dogs (upper GIB, 32; lower GIB, 13; both, 8). MCV was measured in 54 dogs (upper GIB, 33; lower, 13; both, 8), and Hct in 55 dogs (upper GIB, 34; lower GIB, 13; both, 8).

Abbreviations: GIB, gastrointestinal bleeding; Hb, hemoglobin; Hct, hematocrit; MCHC, mean corpuscular hemoglobin concentration; MCV, mean corpuscular volume; UCR, urea nitrogen/creatinine ratio.

^a^ Kruskal‐Wallis test.

Serum urea nitrogen and creatinine concentrations and UCR were not significant predictors of upper GIB (Table 8), even after controlling for the confounding effect of type of GIB (ie, overt vs occult GIB), age, and weight.

**TABLE 8:** Results of logistic regression models examining the association of serum urea nitrogen and creatinine concentrations, UCR, Hb, Hct, MCV, and MCHC and upper GIB compared to lower GIB in dogs

	n	Odds ratio	95% CI	*P* value
Urea nitrogen (mg/dL)	50	0.99^a^	0.94‐1.04	.61
Creatinine (mg/dL)	50	0.37^a^	0.05‐2.70	.33
UCR	50	1.01^a^	0.95‐1.07	.79
		1.01^b^	0.95‐1.06	.82
		1.02^c^	0.95‐1. 09	.60
Hb (g/L)	45	1.01^a^	0.99‐1.03	.26
Hct (L/L)	47	27.17^a^	0.09‐8237	.26
MCV (fL)	46	1.03^a^	0.95‐1.12	.44
		1.01^d^	0.93‐1.09	.89
MCHC (g/L)	45	1.00^a^	0.97‐1.03	.89

*Note:* Thirty‐seven dogs were diagnosed with upper and 13 with lower GIB. Upper GIB was defined as hemorrhage orad to the ligament of Treitz (duodenojejunal junction). Results for Hb and MCHC were available from 45 dogs (upper GIB, 32; lower GIB, 13). MCV was measured in 46 dogs (upper GIB, 33; lower, 13), and Hct in 47 dogs (upper GIB, 34; lower GIB, 13). Confounding effects of age and presence of overt GIB were assessed for all variables. Additionally, confounding effects of weight were assessed for serum creatinine concentration and UCR, and the presence of anorexia and weight loss for serum urea nitrogen and creatinine concentrations, and UCR. If the inclusion of the covariates resulted in a ≥20% change in the coefficient of the independent variable, the adjusted odds ratio was reported.

Abbreviations: CI, confidence interval; GIB, gastrointestinal bleeding; Hb, hemoglobin; Hct, hematocrit; MCHC, mean corpuscular hemoglobin concentration; MCV, mean corpuscular volume; n, sample size; UCR, urea nitrogen/creatinine ratio.

^a^ Adjusted odds ratio after inclusion of type of GIB (overt vs occult) as covariate.

^b^ Adjusted odds ratio after inclusion of weight as covariate.

^c^ Adjusted odds ratio after inclusion of presence of anorexia as covariate.

^d^ Adjusted odds ratio after inclusion of presence of age as covariate.

No significant difference was found among dogs with upper, lower, and both upper and lower GIB for all hematological variables (Table 7). Using logistic regression models, none of the variables were significantly associated with localization of GIB (upper vs lower; Table 8). For each variable, we controlled for the confounding effect of the type of GIB (ie, overt vs occult GIB).

## 4 DISCUSSION

In our study population, UCR was not significantly associated with the type of GIB (occult vs healthy) or the localization of GIB (upper vs lower GIB) in dogs. These results were unexpected.

The first part of our study aimed to investigate UCR as a marker for occult GIB in dogs. Results indicated that UCR of dogs with occult GIB was not significantly different from clinically healthy dogs and was not associated with the odds of having occult GIB. Similar results have been reported previously in a study in which UCR was not significantly increased in human patients with occult upper GIB compared with patients without upper GIB who had upper GI endoscopy performed for various reasons; and UCR had poor discriminatory ability to predict occult upper GIB (area under the curve, 0.605) in humans.^22^ Occult GIB is characterized by microscopic blood loss, whereas clinically relevant hemorrhage causes visible GIB in cases of overt hemorrhage.^23^ People with occult GIB can lose up to 100 mL blood per day representing approximately 22 g of protein loss (7 g plasma protein, 15 g hemoglobin).^24^, ^25^ In a previous study, 180 g of blood protein was instilled in the form of diluted citrated blood into the stomach of healthy men and a 25% to 35% increase in serum urea concentration from baseline was noted.^14^ The amount of protein lost that could be absorbed in occult GIB may be insufficient to cause an increase in serum urea concentration in dogs. Alternatively, occult GIB may not result in prerenal azotemia.

When assessing serum urea nitrogen and creatinine concentrations separately, serum urea nitrogen concentration was significantly lower in occult GI bleeders compared to healthy dogs and compared to overt GI bleeders. Unexpectedly, dogs with a serum urea nitrogen concentration of ≤12 mg/dL were significantly more likely to have occult GIB than were dogs with serum urea nitrogen concentration ranging from >12 to ≤21.5 mg/dL. Anorexia could have blunted a possible increase in serum urea nitrogen concentration and therefore also an increase in UCR secondary to occult GIB. Decreased or absent food intake and therefore decreased protein digestion could lead to lower serum urea nitrogen concentrations, potentially masking increases associated with GIB.^26^ In fact, based on previous study results, serum urea nitrogen concentrations may be more influenced by dietary protein intake than GIB.^14^ Serum creatinine concentrations in dogs with overt and occult GIB were significantly lower compared to healthy control dogs, and the odds of occult GIB compared to being healthy were lower in dogs with higher serum creatinine concentrations. We hypothesized that the cause for decreased serum creatinine concentrations in the dogs with GIB was decreased body weight and muscle mass compared to healthy dogs because it was shown recently that serum creatinine concentration and lean body mass are positively correlated.^27^ Although marked muscle loss was an exclusion criterion, dogs with GIB could have had decreased muscle mass compared to the control dogs. However, even after controlling for the confounding effect of anorexia and weight loss in the logistic regression model, UCR was not significantly associated with presence of occult GIB. This finding may represent type II statistical error because of small sample size or failure to recognize and control another confounding variable.

The sensitivity of UCR to diagnose GIB of any form also may be decreased in our study because the maximum change in serum urea nitrogen concentration could have been missed depending on the timing of the analysis vs timing of hemorrhage. In dogs, the peak serum urea nitrogen concentration occurs approximately 4.5 to 10 hours after blood digestion and serum urea nitrogen concentration decrease to baseline by 24 hours.^12^ The timing of GIB to blood collection may preclude useful findings, particularly in dogs with suspected GIB receiving GI protectants. In the dogs in our study, 42.7% were receiving GI protectants at the time of blood sampling.

In our study, the median UCR in dogs with overt GIB was significantly higher compared to the control group of clinically healthy dogs and occult GI bleeders. Unfortunately, predicting overt GIB is less clinically useful, because overt GIB is not diagnostically challenging. A retrospective study of dogs with hematemesis, melena, or both and control dogs found that serum urea nitrogen and creatinine concentrations and UCR were significantly higher compared to control dogs.^1^ Compared to our results, medians of serum urea nitrogen and creatinine concentrations and UCR in dogs with upper GIB were higher.^1^ Similarly, in a study that assessed dogs with severe upper or lower GI hemorrhage or both requiring blood transfusion, a higher mean UCR of 34 was found when compared to our results where the median UCR for overt GIB was 19.3 (mean, 24.9). This difference may be attributed to differences in inclusion criteria, because patients in shock, moderate to marked dehydration, or both were excluded in our study.

Dogs with occult and overt GIB were significantly older than the healthy dogs we recruited. In a recent study, clinically healthy geriatric dogs (≥12 years) had significantly higher UCR than adult (1 to <8 years) or senior dogs (8 to <12 years) with serum urea concentration being increased and serum creatinine concentration being decreased in geriatric dogs.^28^ Consequently, we included the potential confounding effect of age in our analyses as a possible covariate in the logistic regression models.

Our results may suggest that UCR has poor discriminatory ability to distinguish upper GIB from lower GIB in dogs. Increased UCR was not associated with higher odds of having upper vs lower GIB in our study. This was an unexpected finding, because UCR cutoffs ranging from 30 to 36 are reported as markers for upper GIB in people.^7^, ^8^, ^9^, ^10^, ^11^ These contradictory results may be a result of differences in inclusion criteria. Many studies in humans included only patients with overt GIB,^6^, ^7^, ^29^, ^30^, ^31^ whereas both overt and occult GI bleeders were enrolled in our study. After controlling the type of GIB, an association between location of GIB and UCR was not identified in our study. This may represent type II statistical error because of small patient number, especially for lower GI bleeders. Future studies including only dogs with overt GIB and a larger sample size are warranted.

Another reason for the poor discriminatory ability of UCR regarding localization of the bleeding lesion may be that the distinction into upper and lower GIB represents an anatomical, and not a physiological, classification based on localization of the bleeding lesion either orad or aborad to the ligament of Treitz (duodenojejunal junction). However, because the entire small intestine is considered the major site of amino acid absorption,^32^ jejunal and ileal hemorrhage also may result in absorption of blood protein breakdown products and subsequent hepatic ureagenesis. Finally, although the colon does not play a major role in protein digestion, it has been shown that in dogs it is a location for substantial microbial catabolism of undigested and endogenous nitrogen‐containing compounds, utilization of these compounds for microbial growth, and absorption of ammonia from catabolized amino acids.^33^ Therefore, colonic hemorrhage in dogs could result in increased hepatic production of urea because of absorption of protein breakdown products, ammonia, or both.

When comparing UCR to routine hematological variables, our results show that decreases in Hb and Hct are more useful than UCR in predicting occult GIB. In contrast, MCHC and MCV results were not associated with increased odds of having occult GIB. Similar to our results, another study found that a decrease in Hb was a better predictor of occult upper GIB in humans than UCR.^22^ Given a previous study that showed that Hct and MCV are decreased in clinically healthy geriatric dogs compared to adult dogs,^28^ we assessed if age acted as a confounding variable and reported adjusted OR if that was the case. However, despite including age as a covariate, no significant association was found between MCV or MCHC and the odds of occult GIB.

In our study, Hb and Hct were not significantly different between dogs with upper and lower GIB. This result is consistent with a study that found no significant difference in Hb between upper and lower GIB in children.^30^ However, in contrast to our study, several other studies in people have found that Hb, Hct, or both were significantly decreased in patients with upper GIB compared to those with lower GIB.^5^, ^7^, ^31^ In these studies, it was postulated that upper GIB was more severe than lower GIB, resulting in more severe blood loss. Different inclusion criteria among studies may explain these differences. As stated earlier, the fact that more dogs with lower GIB had overt GIB whereas dogs with upper GIB more frequently had occult hemorrhage could explain our results.

Our present study had several limitations. First, the small number of occult GI bleeders, as well as the low number of dogs with lower GIB, could have decreased overall statistical power and therefore the chance of detecting a true effect. Second, because of the partially retrospective nature of our study, information on diet and systemic blood pressure at the time of blood collection that could have influenced serum urea nitrogen or creatinine concentrations or both was not routinely documented and therefore could not be controlled. Third, additional renal function testing, such as measurement of symmetric dimethylarginine, urinalysis, or both, was not routinely performed in our retrospectively enrolled patients. Therefore, some patients may have had early kidney disease that was not detected by routine laboratory testing. A fourth limitation is that the control dogs were deemed healthy based on history, physical examination findings, and blood test results but did not have additional diagnostic tests performed to rule out occult GIB. Using dogs from the same hospitals with clinical suspicion of occult GIB but confirmed absence of GI hemorrhage would have provided a better control group.

Another possible limitation is that bleeding lesions could have been missed in parts of the GI tract that were not examined. Ideally, only dogs receiving both upper and lower GI endoscopy, complete VCE examination, or both would have been included to minimize misclassification bias. However, doing so would have substantially decreased our sample size. Additionally, because the diagnostic yield of VCE can be decreased by poor visibility of the GI mucosa and incomplete studies,^34^ some dogs with missed bleeding lesions may have been falsely excluded from analysis or could have been misclassified. In human patients with obscure overt and occult GIB, sensitivity and specificity of 88.9% and 95%, respectively, have been reported for VCE.^35^ Nonetheless, studies evaluating the diagnostic sensitivity, specificity, and diagnostic yield of VCE to detect GIB in a large number of dogs are lacking.

Including dogs receiving GI protectants may be a limitation in our study. Successful treatment of gastroduodenal ulceration using protein‐pump inhibitors or other GI protectants could have blunted a possible increase in UCR. Ideally, dogs receiving GI protectants would have been excluded from the study. However, most dogs enrolled retrospectively had been prescribed these medications before referral, and excluding these dogs would have decreased patient enrollment. Additionally, withholding GI protectants in dogs enrolled prospectively despite suspected or confirmed GIB would not have been ethical. Excluding these patients would have resulted in very small sample size, likely precluding sufficient statistical power. We calculated descriptive statistics after excluding dogs that received GI protectants, which produced very similar results compared to the original data (Tables S3 and S4). We therefore believe that the overall potential influence of GI protectants on our results was small.

Another limitation is that hematological and biochemical analyses were performed at different laboratories and using different in‐house analyzers. Therefore, results could have been influenced by interassay and interlaboratory variation especially given the inclusion of samples run on in‐house analyzers, which could have been a source of bias. Only 9 of 154 (5.8%) samples however had in‐house blood analysis performed and, even after exclusion of these, UCR was not associated with occult GIB and could not distinguish between upper and lower GIB (Tables S5‐S8). Finally, hematologic variables only were assessed at a single point in time. Future studies should assess if changes in UCR over time rather than a single result could be helpful in identifying occult GIB.

## 5 CONCLUSION

Our data suggest that the UCR is not useful in predicting occult GIB and does not have strong discriminatory ability to distinguish upper from lower GIB in dog. Consequently, increased UCR in a dog without signs of overt GIB, especially if Hct is within the middle or upper reference interval, does not support prompt prescription of GI protectants. Other factors, such as anorexia, high protein diet, weight loss, muscle condition, and corticosteroid administration should be considered when interpreting UCR.

## CONFLICT OF INTEREST DECLARATION

Alice Defarges has been an employee of Infiniti Medical since March 1, 2020, but did not work for this company during enrollment of patients. Jill Pomrantz was a previous employee of Infiniti Medical. No other authors have a conflict of interest.

## OFF‐LABEL ANTIMICROBIAL DECLARATION

Some dogs in this study received metronidazole, sulfasalazine, tylosin, ampicillin, cefazolin, and doxycycline as part of their treatment for either GI disease (metronidazole, sulfasalazine, tylosin) or extra‐GI disease (ampicillin, cefazolin, and doxycycline). These drugs are not licensed for canine use in Canada and were chosen as they are considered safe and their use in these patients was deemed indicated.

## INSTITUTIONAL ANIMAL CARE AND USE COMMITTEE (IACUC) OR OTHER APPROVAL DECLARATION

For data gathered retrospectively, the authors declare no IACUC or other approval was needed. For data gathered prospectively, the study was approved by the University of Guelph Animal Care Committee, and owner consent was obtained before blood collection and administration of video capsule endoscopy.

## HUMAN ETHICS APPROVAL DECLARATION

Authors declare human ethics approval was not needed for this study.

## Supporting information


**TABLE S1** Biochemistry analyzers used in reference laboratories
**Table S2** Hematology analyzers used in reference laboratories
**Table S3** Descriptive statistics of serum urea nitrogen and creatinine concentrations, UCR, Hb, Hct, MCV, and MCHC between dogs with overt and occult GIB and clinically healthy dogs after exclusion of dogs that received gastrointestinal protectants
**Table S4** Descriptive statistics of serum urea nitrogen and creatinine concentrations, UCR, Hb, Hct, MCV, and MCHC in dogs with upper GIB, lower GIB and with both upper and lower GIB after exclusion of dogs that received gastrointestinal protectants
**Table S5** Comparison of serum urea nitrogen and creatinine concentrations, UCR, Hb, Hct, MCV, and MCHC between dogs with overt and occult GIB and clinically healthy dogs after exclusion of cases with in‐house blood analysis
**Table S6** Results of logistic regression models examining the association of serum urea nitrogen and creatinine concentrations, UCR, Hb, Hct, MCV, and MCHC and the odds of having occult GIB in dogs compared to being clinically healthy after exclusion of cases with in‐house blood analysis
**Table S7** Comparison of serum urea nitrogen and creatinine concentrations, UCR, Hb, Hct, MCV, and MCHC in dogs with upper GIB, lower GIB and with both upper and lower GIB after exclusion of cases with in‐house blood analysis
**Table S8** Results of logistic regression models examining the association of serum urea nitrogen and creatinine concentrations, UCR, Hb, Hct, MCV, and MCHC and upper GIB compared to lower GIB in dogs after exclusion of cases with in‐house blood analysis

## References

[1] Prause LC , Grauer GF . Association of gastrointestinal hemorrhage with increased blood urea nitrogen and BUN/creatinine ratio in dogs: a literature review and retrospective study. Vet Clin Pathol. 1998;27(4):107‐111. 12075537 10.1111/j.1939-165x.1998.tb01028.x

[2] Waldrop JE , Rozanski EA , Freeman LM , Rush JE . Packed red blood cell transfusions in dogs with gastrointestinal hemorrhage: 55 cases (1999‐2001). J Am Anim Hosp Assoc. 2003;39(6):523‐527. 14736715 10.5326/0390523

[3] Fitzgerald E , Barfield D , Lee KC , et al. Clinical findings and results of diagnostic imaging in 82 dogs with gastrointestinal ulceration. J Small Anim Pract. 2017;58(4):211‐218. 28276120 10.1111/jsap.12631

[4] Stanton ME , Bright RM . Gastroduodenal ulceration in dogs. Retrospective study of 43 cases and literature review. J Vet Intern Med. 1989;3(4):238‐244. 2685273 10.1111/j.1939-1676.1989.tb00863.x

[5] Tomizawa M , Shinozaki F , Hasegawa R , et al. Laboratory test variables useful for distinguishing upper from lower gastrointestinal bleeding. World J Gastroenterol. 2015;21(20):6246‐6251. 26034359 10.3748/wjg.v21.i20.6246 PMC4445101

[6] Ernst AA , Haynes ML , Nick TG , Weiss SJ . Usefulness of the blood urea nitrogen/creatinine ratio in gastrointestinal bleeding. Am J Emerg Med. 1999;17(1):70‐72. 9928705 10.1016/s0735-6757(99)90021-9

[7] Witting MD , Magder L , Heins AE , Mattu A , Granja CA , Baumgarten M . ED predictors of upper gastrointestinal tract bleeding in patients without hematemesis. Am J Emerg Med. 2006;24(3):280‐285. 16635697 10.1016/j.ajem.2005.11.005

[8] Felber S , Rosenthal P , Henton D . The BUN/creatinine ratio in localizing gastrointestinal bleeding in pediatric patients. J Pediatr Gastroenterol Nutr. 1988;7(5):685‐687. 3263488 10.1097/00005176-198809000-00011

[9] Prieto Bozano G , Escribano Burgos C , Ortega Páez E , Carrasco Gandía S , Lama More R , Polanco Allue I . Value of the BUN/creatinine ratio in localizing digestive system hemorrhage in children. An Esp Pediatr. 1990;32(3):222‐224. 2346258

[10] Richards RJ , Donica MB , Grayer D . Can the blood urea nitrogen/creatinine ratio distinguish upper from lower gastrointestinal bleeding? J Clin Gastroenterol. 1990;12(5):500‐504. 2229992 10.1097/00004836-199010000-00004

[11] Urashima M , Toyoda S , Nakano T , et al. BUN/Cr ratio as an index of gastrointestinal bleeding mass in children. J Pediatr Gastroenterol Nutr. 1992;15(1):89‐92. 1403455 10.1097/00005176-199207000-00014

[12] Yuile C , Hawkins W . Azotemia due to ingestion of blood proteins. Am J Med Sci. 1941;201:162‐167.

[13] Schiff L , Stevens RJ , Goodman S , Garber E , Lublin A . Observations on the oral administration of citrated blood in man. Am J Dig Dis. 1939;6:597‐602.

[14] Cohn TD , Lane M , Zuckerman S , et al. Induced azotemia in humans following massive protein and blood ingestion and the mechanism of azotemia in gastrointestinal hemorrhage. Am J Med Sci. 1956;231(4):394‐401. 13302213 10.1097/00000441-195604000-00004

[15] Johnson JB . The pathogenesis of azotemia in hemorrhage from the upper gastrointestinal tract. J Clin Invest. 1941;20:161‐168. 16694821 10.1172/JCI101208 PMC435044

[16] Whittemore JC , Mooney AP , Price JM , Thomason J . Clinical, clinicopathologic, and gastrointestinal changes from aspirin, prednisone, or combination treatment in healthy research dogs: a double‐blind randomized trial. J Vet Intern Med. 2019;33(5):1977‐1987. 31397009 10.1111/jvim.15577 PMC6766539

[17] UREAL . Roche Diagnostics GmbH. 2019. http://labogids.sintmaria.be/sites/default/files/files/ureal_2020-01_v13_0.pdf. Accessed January 10, 2021.

[18] VetScan® Comprehensive Diagnostic Profile . Abaxis, Inc. 2002. https://vet.abaxis.co.uk/wp-content/uploads/2015/01/VetScan-Comprehensive-Diagnostic-Profile-PI.pdf. Accessed January 10, 2021.

[19] Urea Nitrogen . Beckman Coulter Inc. 2009. https://www.beckmancoulter.com/wsrportal/techdocs?docname=/cis/BAOSR6X34/%%/EN_UREA_BAOSR6x34.pdf. Accessed January 10, 2021.

[20] Feldman EC , Nelson RW , Reusch CE , et al. Canine and Feline Endocrinology. 4th ed. St. Louis, MO: Elsevier Saunders; 2015.

[21] Dohoo I , Martin W , Stryhn H . Veterinary Epidemiologic Research. 2nd ed. Charlottetown, Prince Edward Island, Canada: VER Inc.; 2010.

[22] Tomizawa M , Shinozaki F , Hasegawa R , et al. Reduced hemoglobin and increased C‐reactive protein are associated with upper gastrointestinal bleeding. World J Gastroenterol. 2014;20(5):1311‐1317. 24574805 10.3748/wjg.v20.i5.1311 PMC3921513

[23] Naut ER . The approach to occult gastrointestinal bleed. Med Clin North Am. 2016;100(5):1047‐1056. 27542424 10.1016/j.mcna.2016.04.013

[24] Rockey DC . Occult gastrointestinal bleeding. Gastroenterol Clin North Am. 2005;34(4):699‐718. 16303578 10.1016/j.gtc.2005.08.010

[25] Ahlquist D . Approach to the patient with occult gastrointestinal bleeding. In: Yamada T , ed. Textbook of Gastroenterology. Vol 1. Philadelphia: J. B. Lippincott; 1995:699‐717.

[26] Tripathi NK , Gregory CR , Latimer KS . Urinary system. In: Latimer KS , ed. Duncan & Prasse's Veterinary Laboratory Medicine: Clinical Pathology. 5th ed. Chichester, UK: Wiley‐Blackwell; 2011:274‐278.

[27] Hall JA , Yerramilli M , Obare E , Yerramilli M , Melendez LD , Jewell DE . Relationship between lean body mass and serum renal biomarkers in healthy dogs. J Vet Intern Med. 2015;29(3):808‐814. 25913398 10.1111/jvim.12607 PMC4895404

[28] Radakovich LB , Pannone SC , Truelove MP , Olver CS , Santangelo KS . Hematology and biochemistry of aging‐evidence of "anemia of the elderly" in old dogs. Vet Clin Pathol. 2017;46(1):34‐45. 28195648 10.1111/vcp.12459

[29] Snook JA , Holdstock GE , Bamforth J . Value of a simple biochemical ratio in distinguishing upper and lower sites of gastrointestinal haemorrhage. Lancet. 1986;1(8489):1064‐1065. 2871338 10.1016/s0140-6736(86)91332-2

[30] Kim KS , Kang CH , Kim JY . Availability of blood urea nitrogen/creatinine ratio in gastrointestinal bleeding with melena in children. Pediatr Gastroenterol Hepatol Nutr. 2015;18(1):30‐38. 25866731 10.5223/pghn.2015.18.1.30 PMC4391998

[31] Sittichanbuncha Y , Senasu S , Thongkrau T , et al. How to differentiate sites of gastrointestinal bleeding in patients with hematochezia by using clinical factors? Gastroenterol Res Pract. 2013;2013:265076. 24348531 10.1155/2013/265076 PMC3852082

[32] Hall EJ , Day MJ . Diseases of the small intestine. In: Ettinger SJ , Feldman EC , Côté E , eds. Textbook of Veterinary Internal Medicine. Vol 2. St. Louis, Missouri: Elsevier; 2017:1516‐1518.

[33] Hendriks WH , van Baal J , Bosch G . Ileal and faecal protein digestibility measurement in humans and other non‐ruminants—a comparative species view. Br J Nutr. 2012;108(Suppl 2):S247‐S257. 23107535 10.1017/S0007114512002395

[34] Davignon DL , Lee AC , Johnston AN , et al. Evaluation of capsule endoscopy to detect mucosal lesions associated with gastrointestinal bleeding in dogs. J Small Anim Pract. 2016;57(3):148‐158. 26931500 10.1111/jsap.12442

[35] Pennazio M , Santucci R , Rondonotti E , et al. Outcome of patients with obscure gastrointestinal bleeding after capsule endoscopy: report of 100 consecutive cases. Gastroenterology. 2004;126(3):643‐653. 14988816 10.1053/j.gastro.2003.11.057

