# Supplementary material for: Diagnostic evaluation of urea nitrogen/creatinine ratio in dogs with gastrointestinal bleeding
Source: J Vet Intern Med. 2021 Mar 17;35(3):1427–38. doi: 10.1111/jvim.16101 (PMC8162593; doi:10.1111/jvim.16101)
Supplement: Supplementary file 1 — TABLE S1 Biochemistry analyzers used in reference laboratories Table S2 Hematology analyzers used in reference laboratories Table S3 Descriptive statistics of serum urea nitrogen and creatinine concentrations, UCR, Hb, Hct, MCV, and MCHC between dogs with overt and occult GIB and clinically healthy dogs after exclusion of dogs that received gastrointestinal protectants Table S4 Descriptive statistics of serum urea nitrogen and creatinine concentrations, UCR, Hb, Hct, MCV, and MCHC in dogs with upper GIB, lower GIB and with both upper and lower GIB after exclusion of dogs that received gastrointestinal protectants Table S5 Comparison of serum urea nitrogen and creatinine concentrations, UCR, Hb, Hct, MCV, and MCHC between dogs with overt and occult GIB and clinically healthy dogs after exclusion of cases with in‐house blood analysis Table S6 Results of logistic regression models examining the association of serum urea nitrogen and creatinine concentrations, UCR, Hb, Hct, MCV, and MCHC and the odds of having occult GIB in dogs compared to being clinically healthy after exclusion of cases with in‐house blood analysis Table S7 Comparison of serum urea nitrogen and creatinine concentrations, UCR, Hb, Hct, MCV, and MCHC in dogs with upper GIB, lower GIB and with both upper and lower GIB after exclusion of cases with in‐house blood analysis Table S8 Results of logistic regression models examining the association of serum urea nitrogen and creatinine concentrations, UCR, Hb, Hct, MCV, and MCHC and upper GIB compared to lower GIB in dogs after exclusion of cases with in‐house blood analysis [file JVIM-35-1427-s001.pdf]

SUPPLEMENTARY DATA

TABLE S1 Biochemistry analyzers used in reference laboratories

**Reference laboratory**	**Biochemistry analyzer**	**Number of samples**
Animal Health Laboratory, University of Guelph, Guelph, Canada	• Cobas c501 (Roche Diagnostics Canada, Laval, Canada)	• 117 (42 overt GIB, 12 occult GIB, 63 healthy)
	• Vetscan HM5 (Abaxis Veterinary Diagnostics, Union City, California)	• 7 (5 overt GIB 2 occult GIB)
IDEXX Reference Laboratories Ltd., various locations	• Beckman Coulter AU5812 Clinical Chemistry Analyzer (Beckman Coulter Canada, LP., Mississauga, Canada)	• 15 (8 overt GIB, 7 occult GIB)
Antech Diagnostics Reference Laboratories, various locations	• Chemistry Analyzer AU2700 (Olympus Canada Inc., Richmond Hill, Canada)	• 3 (overt GIB)

Notes: For 3 dogs (2 overt, 1 occult), information on the used instruments for biochemistry analysis was not available.

Abbreviation: GIB, gastrointestinal bleeding.

TABLE S2 Hematology analyzers used in reference laboratories

Reference laboratory	Hematology analyzer	Number of samples
Animal Health Laboratory, University of Guelph, Guelph, Canada	• ADVIA 2120 (Siemens Canada, Mississauga, Canada)	• 124 (46 overt GIB, 19 occult GIB, 63 healthy)
IDEXX Reference Laboratories Ltd., various locations	• Sysmex XN-9100-300 Automated Hematology Analyzer (Sysmex Canada, Mississauga, Canada)	• 8 (7 overt GIB, 1 occult GIB)
Antech Diagnostics Reference Laboratories, various locations	• ADVIA 2120 (Siemens Canada, Mississauga, Canada)	• 3 (overt GIB)

Notes: Hematology analysis was not performed in 4 dogs (2 overt, 2 occult). For 3 dogs (2 overt, 1 occult), information on the used instruments for hematology analysis was not available.

Abbreviation: GIB, gastrointestinal bleeding.

TABLE S3 Descriptive statistics of serum urea nitrogen and creatinine concentrations, UCR, Hb, Hct, MCV, and MCHC between dogs with overt and occult GIB and clinically healthy dogs after exclusion of dogs that received gastrointestinal protectants

	**Overt GIB (n = 39)**	**Occult GIB (n = 13)**	**Healthy (n = 65)**
**Urea nitrogen** (mg/dL)	17.6 (6.2 – 51.5)	12.3 (5.6 – 41.5)	17.4 (9.2 – 29.4)
**Creatinine** (mg/dL)	0.85 (0.38 – 1.92)	0.85 (0.50 – 1.22)	1.04 (0.31 – 1.66)
**UCR**	19.4 (9.8 – 73.2)	14.5 (9.2 – 43.1)	16.2 (8.0 – 47.7)
**Hb** (g/L)	136.3 ± 47.0	124.2 ± 43.5	174.5 ± 17.09
**Hct** (L/L)	0.41 ± 0.13	0.36 ± 0.12	0.51 ± 0.05
**MCV** (fL)	70 (54 – 79)	72.5 (49.0 – 88.6)	71 (64 – 78)
**MCHC** (g/L)	335 ± 16	342 ± 30	340 ± 9

Note: The non-normally distributed data are expressed as median (range). Normally-distributed data were expressed as mean ± standard deviation.

Abbreviations: GIB, gastrointestinal bleeding; Hb, hemoglobin; Hct, hematocrit; MCHC, mean corpuscular hemoglobin concentration; MCV, mean corpuscular volume; UCR, urea nitrogen/creatinine ratio.

TABLE S4 Descriptive statistics of serum urea nitrogen and creatinine concentrations, UCR, Hb, Hct, MCV, and MCHC in dogs with upper GIB, lower GIB and with both upper and lower GIB after exclusion of dogs that received gastrointestinal protectants

	**Upper GIB (n = 15)**	**Lower GIB (n = 9)**	**Both (n = 4)**
**Urea nitrogen** (mg/dL)	15.1 (5.6 – 41.5)	14.3 (9.5 – 51.5)	17.9 (15.4 – 20.2)
**Creatinine** (mg/dL)	0.96 (0.5 – 1.22)	0.85 (0.38 – 1.92)	0.89 (0.8 – 1.2)
**UCR**	16.3 (9.2 – 43.1)	24.8 (10.4 – 36.3)	19.2 (15.8 – 22.3)
**Hb** (g/L)	141.5 (35 – 179)	155 (43 – 179)	158.5 (109 – 202)
**Hct** (L/L)	0.39 ± 0.11	0.36 ± 0.15	0.48 ± 0.12
**MCV** (fL)	71 (49 – 81)	69 (59 – 88.6)	73 (63 – 79)
**MCHC** (g/L)	342 ± 20	332 ± 30	325 ± 17

Notes: The non-normally distributed data are expressed as median (range). Normally-distributed data were expressed as mean ± standard deviation.

Abbreviations: GIB, gastrointestinal bleeding; Hb, hemoglobin; Hct, hematocrit; MCHC, mean corpuscular hemoglobin concentration; MCV, mean corpuscular volume; UCR, urea nitrogen/creatinine ratio.

TABLE S5 Comparison of serum urea nitrogen and creatinine concentrations, UCR, Hb, Hct, MCV, and MCHC between dogs with overt and occult GIB and clinically healthy dogs after exclusion of cases with in-house blood analysis

	**Overt GIB**	**Occult GIB**	**Healthy**	***P* value**
**Urea nitrogen** (mg/dL)	17.3 (4.2 – 71)[d]	12.4 (5.6 – 44.5)[d]	16.8 (9.2 – 29.4)	.03[a]
**Creatinine** (mg/dL)	0.88 ± 0.30[d]	0.82 ± 0.30[e]	1.03 ± 0.24[d,e]	.001[b]
**UCR**	19.3 (8.1 – 89.4)[d]	15.3 (7.2 – 75.7)	16.0 (8.0 – 47.7)[d]	.01[c]
**Hb** (g/L)	146 (36 – 202)[d]	127 (35 – 179)[e]	176 (140 – 228)[d,e]	< .0001[c]
**Hct** (L/L)	0.43 (0.13 – 0.59)[d]	0.36 (0.11 – 0.53)[e]	0.51 (0.42 – 0.65)[d,e]	< .0001[c]
**MCV** (fL)	70 ± 5	67± 12	71 ± 3	.38[c]
**MCHC** (g/L)	332 (253 – 372)[d]	337 (277 – 396)	340 (323 – 366)[d]	.001[c]

Notes: Sixty dogs had overt and 22 dogs had occult GIB; 63 dogs were clinically healthy. The non-normally distributed data are expressed as median (range). Normally-distributed data were expressed as mean ± standard deviation. For dogs with overt GIB, Hb, Hct, MCV, and MCHC results were available for 58 dogs. For dogs with occult GIB, Hb and MCHC results were available for 20, Hct and MCV for 21 dogs.

Abbreviations: GIB, gastrointestinal bleeding; Hb, hemoglobin; Hct, hematocrit; MCHC, mean corpuscular hemoglobin concentration; MCV, mean corpuscular volume; UCR, urea nitrogen/creatinine ratio.

[a]ANOVA on logarithmically transformed data.

[b]ANOVA.

[c]Kruskal-Wallis test.

[d/e]Post hoc test revealed significant difference between the groups marked with the same letter.

TABLE S6 Results of logistic regression models examining the association of serum urea nitrogen and creatinine concentrations, UCR, Hb, Hct, MCV, and MCHC and the odds of having occult GIB in dogs compared to being clinically healthy after exclusion of cases with in-house blood analysis

	n	Odds ratio	95% CI	*P* value
**Urea nitrogen** (mg/dL)	85	-	-	-
**1. Quartile** (5 > Urea nitrogen ≤ 12)	22	(Referent)	-	-
**2. Quartile** (12 > Urea nitrogen ≤ 16)	23	0.07[a]	0.01 – 0.66	.02
**3. Quartile** (16 > Urea nitrogen ≤ 21.5)	20	0.08[a]	0.01 – 0.70	.02
**4. Quartile** (21.5 > Urea nitrogen≤ 45)	20	0.38[a]	0.06 – 1.28	.10
**Creatinine** (mg/dL)	85	0.04	0.01 – 0.33	.003
**UCR**	85	1.03[a]	0.98 – 1.08	.27
		1.03[b]	0.96 – 1.11	.40
**Hb** (g/L)	83	0.90	0.86 – 0.95	.0001
**Hct** (L/L) per 100 units	84	0.70	0.58 – 0.84	.0001
**MCV** (fL)	84	-	-	-
**1. Quartile** (37 > MCV ≤ 68)	22	(Referent)	-	-
**2. Quartile** (68 > MCV ≤ 71)	26	0.49[c]	0.13 – 1.88	.30
**3. Quartile** (71 > MCV ≤ 73)	20	0.22[c]	0.04 – 1.18	.08
**4. Quartile** (73 > MCV ≤ 89)	16	1.16[c]	0.31 – 4.81	.78
**MCHC** (g/L)	83	-	-	-
**1. Quartile** (276 > MCHC ≤ 332)	22	(Referent)	-	-
**2. Quartile** (332 > MCHC ≤ 339)	20	0.77[c]	0.21 – 2.84	.70
**3. Quartile** (339 > MCHC ≤ 346)	26	0.25[c]	0.06 – 1.15	.08
**4. Quartile** (346 > MCHC ≤ 396)	15	0.50[c]	0.10 – 2.41	.39

Notes: Twenty-two dogs had occult GIB; 63 dogs were clinically healthy. For dogs with occult GIB, Hb and MCHC were available for 20, Hct and MCV for 21 dogs. Serum urea nitrogen concentration, MCV, and MCHC were modeled as categorical variable to meet the assumption of linearity. Confounding effect of age was assessed for all variables, the effect of weight was assessed for serum creatinine concentration and UCR, and the presence of anorexia and weight loss for serum urea nitrogen and creatinine concentrations, and UCR. If the inclusion of the covariates resulted in a ≥ 20% change in the coefficient of the independent variable, the adjusted odds ratio was reported.

Abbreviations: CI, confidence interval; GIB, gastrointestinal bleeding; Hb, hemoglobin; Hct, hematocrit; MCHC, mean corpuscular hemoglobin concentration; MCV, mean corpuscular volume; n, sample size; UCR, urea nitrogen/creatinine ratio.

[a]adjusted odds ratio after inclusion of presence of weight loss as covariate.

[b]adjusted odds ratio after inclusion of presence of anorexia as covariate.

[c]adjusted odds ratio after inclusion of age as covariate.

TABLE S7 Comparison of serum urea nitrogen and creatinine concentrations, UCR, Hb, Hct, MCV, and MCHC in dogs with upper GIB, lower GIB and with both upper and lower GIB after exclusion of cases with in-house blood analysis

	**Upper GIB**	**Lower GIB**	**Both**	***P* value**[a]
**Urea nitrogen** (mg/dL)	15.7 (5.6 – 71.0)	18.8 (9.5 – 51.5)	17.9 (10.6 – 35.8)	.35
**Creatinine** (mg/dL)	0.87 ± 0.31	0.98 ± 0.39	0.96 ± 0.19	.60
**UCR**	17.0 (7.2 – 75.7)	22.41 (10.4 – 36.3)	19.2 (11.7 – 37.3)	.60
**Hb** (g/L)	141.5 (35 – 201)	155 (43 – 192)	118.5 (36 – 202)	.92
**Hct** (L/L)	0.4 ± 0.11	0.37 ± 0.16	0.39 ± 0.17	.93
**MCV** (fL)	70 (38 – 81)	69 (41.7 – 88.6)	73 (58 – 79)	.59
**MCHC** (g/L)	333 ± 21	328 ± 26	317 ± 30	.37

Notes: Thirty-five dogs were diagnosed with upper and 11 with lower GIB; 8 dogs had both upper and lower GIB. Upper GIB was defined as hemorrhage orad to the ligament of Treitz (duodenojeunal junction). The non-normally distributed data are expressed as median (range). Normally-distributed data were expressed as mean ± standard deviation. Results for Hb and MCHC were available from 51 dogs (upper GIB, 32; lower GIB, 11; both, 8). Hematocrit and MCV were measured in 52 dogs (upper GIB, 33; lower GIB, 11; both, 8).

Abbreviations: GIB, gastrointestinal bleeding; Hb, hemoglobin; Hct, hematocrit; MCHC, mean corpuscular hemoglobin concentration; MCV, mean corpuscular volume; UCR, urea nitrogen/creatinine ratio.

[a]Kruskal-Wallis test.

TABLE S8 Results of logistic regression models examining the association of serum urea nitrogen and creatinine concentrations, UCR, Hb, Hct, MCV, and MCHC and upper GIB compared to lower GIB in dogs after exclusion of cases with in-house blood analysis

	n	Odds ratio	95% CI	*P* value
**Urea nitrogen**(mg/dL)	46	1.00[a]	0.94 – 1.05	.89
**Creatinine** (mg/dL)	46	0.57[a]	0.07 – 4.79	.60
		0.29[b]	0.04 – 2.35	.25
		0.30[c]	0.03 – 2.67	.28
**UCR**	46	1.01[a]	0.94 – 1.08	.83
		1.01[b]	0.96 – 1.08	.65
		1.00[c]	0.95 – 1. 06	.99
		1.01[d]	0.94 – 1.07	.88
**Hb** (g/L)	43	1.01[a]	1.00 – 1.03	.12
**Hct** (L/L) per 100 units	44	1.06[a]	0.99 – 1.23	.11
**MCV** (fL)	44	1.05[a]	0.96 – 1.16	.28
**MCHC** (g/L)	43	1.02[a]	0.98 – 1.06	.42

Notes: Thirty-five dogs were diagnosed with upper and 11 with lower GIB. Upper GIB was defined as hemorrhage orad to the ligament of Treitz (duodenojejunal junction). Results for Hb and MCHC were available from 43 dogs (upper GIB, 32; lower GIB, 11). Hematocrit and MCV were available in 44 dogs (upper GIB, 33; lower, 11). Confounding effects of age and presence of overt GIB were assessed for all variables. Additionally, confounding effects of weight was assessed for serum creatinine concentration and UCR, and presence of anorexia and weight loss for serum urea nitrogen and creatinine concentrations, and UCR. If the inclusion of the covariates resulted in a ≥ 20% change in the coefficient of the independent variable, the adjusted odds ratio was reported.

Abbreviations: CI, confidence interval; GIB, gastrointestinal bleeding; Hb, hemoglobin; Hct, hematocrit; MCHC, mean corpuscular hemoglobin concentration; MCV, mean corpuscular volume; n, sample size; UCR, urea nitrogen/creatinine ratio.

[a]adjusted odds ratio after inclusion of type of GIB (overt versus occult) as covariate.

[b]adjusted odds ratio after inclusion of weight as covariate.

[c]adjusted odds ratio after inclusion of presence of weight loss as covariate.

[b]adjusted odds ratio after inclusion of presence of anorexia as covariate.
